# DEXAMETHASONE–CYCLOPHOSPHAMIDE PULSE THERAPY IN PROGRESSIVE SYSTEMIC SCLEROSIS

**DOI:** 10.4103/0019-5154.70697

**Published:** 2010

**Authors:** Vishalakshi Viswanath, Amey D Sonavane, Aditi C Doshi, Mrunal G Parab

**Affiliations:** *From the Department of Dermatology, Rajiv Gandhi Medical College and Chhatrapati Shivaji Maharaj Hospital, Kalwa, Thane, Maharashtra, India. E-mail: amey_max@yahoo.com*

Sir,

Progressive Systemic Sclerosis (PSS) is a multisystem disorder characterized by vascular abnormalities, connective tissue sclerosis, atrophy and the presence of autoantibodies that result in fibrosis and vascular abnormalities.[1] Symptomatic therapy is the mainstay and immunosuppressive therapies have shown encouraging results in halting disease progression. A pilot study was conducted to determine the efficacy of Dexamethasone-Cyclophosphamide Pulse therapy (DCP) without intermittent cyclophosphamide for a fixed duration of 12 months. The primary aim was to determine whether fewer and fixed number of pulses could arrest or potentially reverse disease progression (cutaneous and pulmonary symptoms).

Fourteen cases (13 females, one male) between 15 to 50 years, with both cutaneous and respiratory symptoms, fulfilling the ARA scleroderma criteria[1] were selected. Pregnant/lactating women were excluded and female patients were advised contraceptive precautions during therapy. Informed consent, detailed history, general and cutaneous examination of each patient was performed.

Median duration of disease was two years (range: eight months - six years). Cutaneous examination revealed Raynaud’s phenomenon and skin tightening (all cases), sclerodactyly (five), hyperpigmentation (four), digital ulceration (three), telangiectasia and calcinosis cutis (two). Exertional dyspnoea (ten), joint involvement (four) and dysphagia (three) were also seen.

Complete hemogram, blood sugar, chest X-ray, liver and renal functions tests (LFTs and RFTs) were done. Specific investigations like pulmonary function tests (PFTs) {10 cases: severe combined restrictive-obstructive pattern (two), severe restrictive pattern (two), moderate restrictive pattern (two), mild restrictive pattern (three), normal PFT (one)}, ANA (five cases: two positive), dsDNA (five cases: two positive) were performed. Each patient received 100 mg dexamethasone in 500 ml, 5% dextrose over three hours for three consecutive days, along with 500 mg cyclophosphamide on the first day, once a month for 12 consecutive months. No intermittent cyclophosphamide or any other immunosuppressant was administered. Vasodilators (six cases) and penicillamine (two cases with severe skin tightening) were co-administered.

Subjective assessment by physicians and patients (grades: mild/moderate/severe) during follow-up showed improvement in Raynaud’s phenomenon in three cases after the first pulse, six after the second and the rest after three pulses. Skin tightening showed improvement in nine cases within five pulses and the rest after eight pulses. Dyspnoea improved in eight of 10 cases within three pulses. Side effects like weight gain (six), backache (two), Cushingoid features (two) and urinary tract infections (seven cases - treated with appropriate antibiotics) were observed. Of the eight cases of repeat PFTs done at the end of therapy, six demonstrated modest improvement in FVC, FEV_1_ and TLC values. There were no significant alterations in blood pressure (monitored monthly), laboratory parameters like routine hemogram and blood sugar (followed up after every pulse) and RFTs and LFTs (at 3,6,12 months).

Sole Dexamethasone pulse therapy (12 – 21 months duration) for PSS has been studied by Pasricha *et al*., Gupta and Ahmad *et al*.[2,3,4] wherein improvement in dyspnoea required 6 - 18 pulses as compared to three pulses in this study. A shorter duration therapy (six months) used by Sharada *et al*. induced a definite clinical improvement, though confined to the skin alone.[5] Airò *et al*. concluded that intravenous cyclophosphamide alone given for six months can achieve a significant improvement in PFTs in patients with active alveolitis.[6] Barbara *et al*. also recorded lung function improvement in 103 patients with alveolitis who received daily as well as pulse cyclophosphamide for 12-18 months.[7] A single pediatric patient studied by Vatwani *et al*. found eight DCPs without intermittent cyclophosphamide to be effective.[8]

Long term outcome and comparative studies are needed to determine the efficacy of therapy in PSS, but the slow progressive nature of disease makes it practically difficult. DCP (without intermittent cyclophosphamide), offers a regimen of fixed and fewer pulses leading to better patient compliance, decreased side effects and early reversal of the cutaneous and pulmonary complaints as compared to sole dexamethasone/cyclophosphamide pulse therapies.

## References

[1] Goodfield MJD, Jones SK, Veale DJ, Burns T, Breathnach S, Cox N, Griffiths C (2004). Systemic Sclerosis. Rook’s Textbook of Dermatology.

[2] Pasricha JS, Ramam M, Shah S (1990). Reversal of systemic sclerosis with dexamethasone pulses. Indian J Dermatol Venereol Leprol.

[3] Gupta R (2003). Systemic sclerosis treated with dexamethasone pulse. Indian J Dermatol Venereol Leprol.

[4] Ahmad QM, Hassan I, Majid I (2003). Evaluation of dexamethasone pulse therapy in systemic sclerosis. Indian J Dermatol Venereol Leprol.

[5] Sharada B, Kumar A, Kakker R, Adya CM, Pande I, Uppal SS (1994). Intravenous dexamethasone pulse therapy in diffuse systemic sclerosis. A randomized placebo-controlled study. Rheumatol Int.

[6] Airò P, Danieli E, Parrinello G, Antonioli CM, Cavazzana I, Toniati P (2004). Intravenous cyclophosphamide therapy for systemic sclerosis. A single-center experience and review of the literature with pooled analysis of lung function test results. Clin Exp Rheumatol.

[7] White B, Moore WC, Wigley FM, Xiao HQ, Wise RA (2000). Cyclophosphamide is associated with pulmonary function and survival benefit in patients with scleroderma and alveolitis. Ann Intern Med.

[8] Vatwani V, Palta SC, Verma N, Pathak PR, Singh RP (1994). Pulse therapy in scleroderma. Indian Pediatr.

